# Aortic annulus rupture after transcatheter aortic valve replacement: successful management of a dangerous complication

**DOI:** 10.1186/s13019-023-02426-8

**Published:** 2023-11-13

**Authors:** Andrew Jones, Hossein Amirjamshidi, Peter Knight, Frederick S. Ling, Kazuhiro Hisamoto

**Affiliations:** 1 University of Rochester School of Medicine and Dentistry, Rochester, NY USA; 2 Division of Cardiac Surgery, Department of Surgery, University of Rochester Medical Center, 601 Elmwood Ave, Box: Surg, Rochester, NY 14627 USA; 3 Division of Cardiology, University of Rochester Medical Center, Rochester, NY USA

**Keywords:** Transcatheter aortic valve replacement, Annulus rupture, Complication, Survival, Surgical aortic valve replacement

## Abstract

**Objective:**

Aortic annulus rupture remains one of the most fatal complications of TAVR. While attempts have been made to describe and predict this complication, the data remains insufficient without evidence-based guidelines for management of this rare complication.

**Methods:**

Here we describe a series of 3 aortic annulus ruptures after TAVR which were managed successfully to hospital discharge.

**Results:**

Patient 1 suffered annulus rupture during balloon valvuloplasty prior to TAVR. The patient became hypotensive, and echocardiogram showed pericardial effusion. The patient underwent pericardiocentesis which transiently improved the blood pressure, but bleeding continued. The patient was transitioned to an open surgical aortic valve replacement due to ongoing hemorrhage. The chest was left open with delayed closure on post-op day 2. The patient was discharged on post-op day 15. Patient 2 was undergoing TAVR valve expansion. The patient became hypotensive. An echocardiogram revealed pericardial effusion. Pericardiocentesis yielded 200 mL of blood. SURGIFLO (Johnson & Johnson Wound Management, Somerville, NJ) was injected into the pericardial space. Aortic root angiography confirmed no further contrast extravasation. A pericardial drain was left in place for 2 days, and the patient was discharged on post-op day 7. Patient 3 received a TAVR valve and post-placement dilation due to paravalvular leak. The echocardiogram showed a pericardial effusion. Pericardiocentesis was performed, yielding 500 cc of blood. The patient’s healthcare proxy declined emergent surgery; thus, a pericardial drain was placed. No hemostatic agents were used, and drainage reduced over several hours. The drain was removed on post-op day 3, and the patient was discharged on post-op day 8.

**Conclusions:**

Based on the timelines in these three cases, and interventions used, the following steps may be employed in the event of annulus rupture: identification of hemodynamic instability, echocardiogram to confirm pericardial effusion, emergent pericardiocentesis, pericardial drain placement for evacuation of the pericardial space and use of hemostatic agents, repeat aortogram to rule out ongoing extravasation. If hemostasis is unable to be achieved and/or the patient becomes hemodynamically unstable at any point, rapid transition to emergent surgical management is necessary. This management strategy proved successful for this case series and warrants further investigation.

## Background

Transcutaneous aortic valve replacement has grown in popularity and scope. With expanding indications for TAVR, care must be taken to reassess for risk of complications in new populations. While this procedure has offered a solution for patients who are not candidates for surgical aortic valve replacement, thus far several case studies present cases of aortic annulus rupture after TAVR. This known complication is rare, with some estimates putting the risk below 1%, but has an estimated case fatality rate greater than 50% [1]. It has been shown to be more strongly associated with bicuspid aortic valve physiology than tricuspid aortic valves, suggesting that underlying patient characteristics and anatomy may play a role in making a patient’s aortic annulus more prone to rupture. Similarly, models have suggested particular patterns of calcification, particularly on the noncoronary leaflet (in tricuspid aortic valves) [2, 3], porcelain aorta [4], and oversizing of the replacement valve are factors in predicting rupture [5]. Other studies have described the epidemiologic outcomes of TAVR rupture [4, 6–10] and some have even undertaken computer models of aortic annular ruptures [3, 11] and proposed theoretical mechanisms of valve annulus rupture [12]. Unfortunately, there are several case series demonstrating aortic annular ruptures that have none of the identified risk factors [6], suggesting there remain undescribed variables that influence aortic annulus rupture. Similarly, little research has investigated the management of patients after rupture; the role of surgical aortic valve replacement (SAVR) or more conservative management has not yet been clarified. Here we present a series of 3 cases of successful management of aortic annulus rupture after TAVR placement, the complete count from a single institution between 2016 and 2022 to add to the growing literature describing the population of patients experiencing this severe complication and the management strategies.

## Case presentation

### Patient 1

**Fig. 1:**
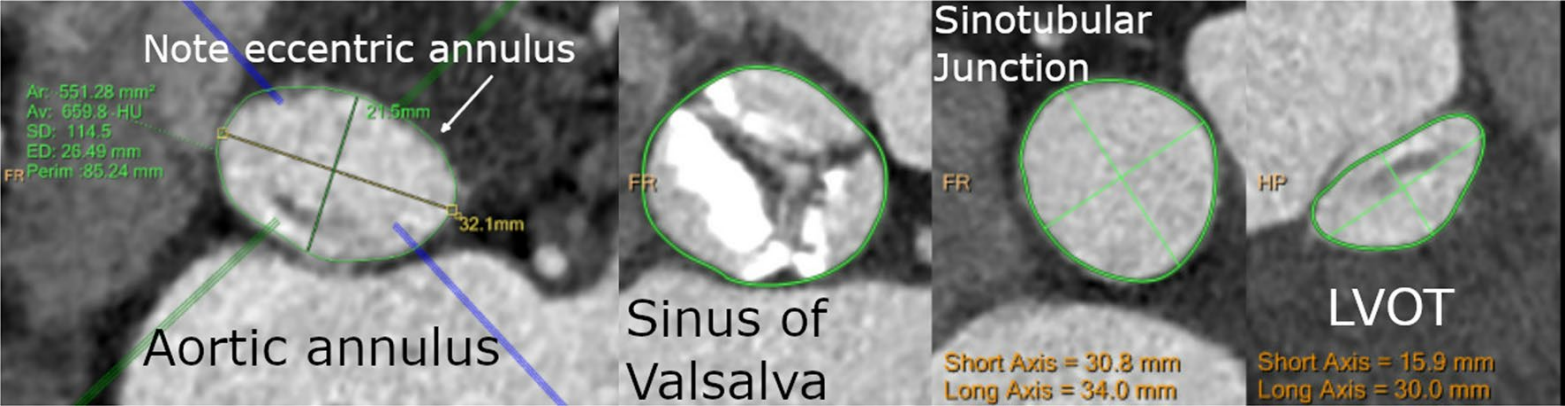
Patient 1 pre-Op CT scan

84 year old female with a BMI of 23.1 and a history significant for coronary artery disease, stage 3 CKD, hypertension, hyperlipidemia, and a 16 pack-year history of smoking who presented for placement of a balloon expandable TAVR, size 29. Her preoperative aortic annulus measurements on CT scan were as follows: Short axis 22.6 mm, long axis 32.5 mm, area defined effective diameter 26.9, square area 570 mm^2^. (Fig. 1) She was noted to have severe calcification of the aortic valve annulus. She was undergoing a subclavian approach when her annulus ruptured during balloon valvuloplasty prior to TAVR. The extravasation of contrast was not initially noted, but she rapidly became hypotensive within 5 min of valvuloplasty. An emergent echocardiogram was performed, and she was noted to have a pericardial effusion. She underwent a pericardiocentesis within 3 min of the initial hypotension event, which yielded blood, and transiently improved the blood pressure, but hemostasis was unable to be achieved even after reversal of anticoagulation. After 9 min of attempting hemostatic control, the decision was made to do an emergent sternotomy with surgical aortic valve replacement. 13 min after the initial hypotension event, a sternotomy was performed, and after 17 min from the initial hypotension event, the patient was placed on cardiopulmonary bypass. She underwent a surgical aortic valve replacement with a 23 mm bioprosthetic valve. She was noted on surgical opening to have damage to both the septum, inferior to the membranous septum, and annulus along the noncoronary sinus, as well as hematoma under the epicardial fat. Hemashield (Maquet, Wayne, NJ) patches were used to close the aorta and septum. Her chest was left open following the procedure with delayed closure on post-op day 2, with one pleural drain, and one mediastinal drain in place. She had multiple episodes of paroxysmal atrial fibrillation throughout her stay that ultimately resolved without further intervention. The remainder of her post-operative care was standard surgical aortic valve replacement care and was without complications. She was discharged to a skilled nursing rehabilitation facility on post-op day 15 and later returned home. At nearly one year follow-up, her surgical aortic valve was functioning normally, with no long term effusion.

### Patient 2

**Fig. 2:**
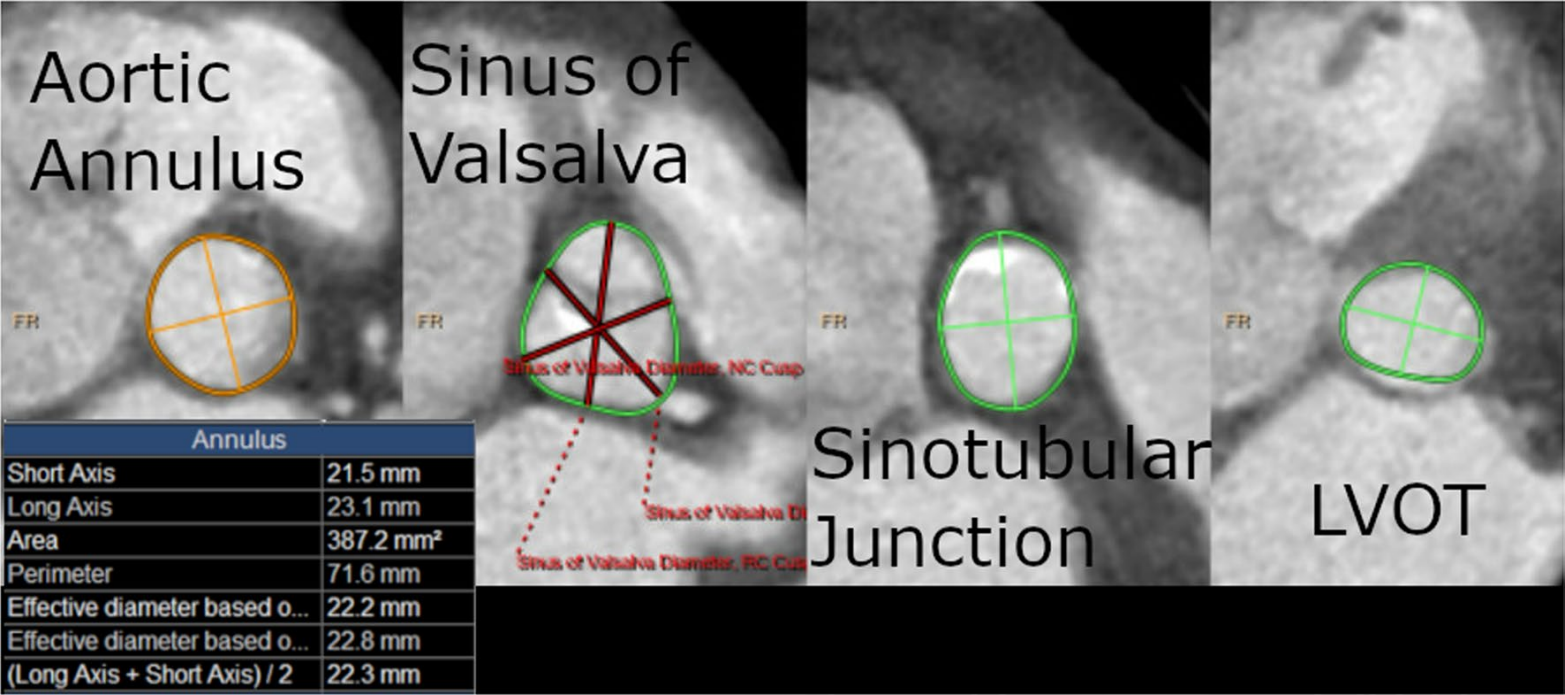
Patient 2 pre-operative CT scan

**Fig. 3:**
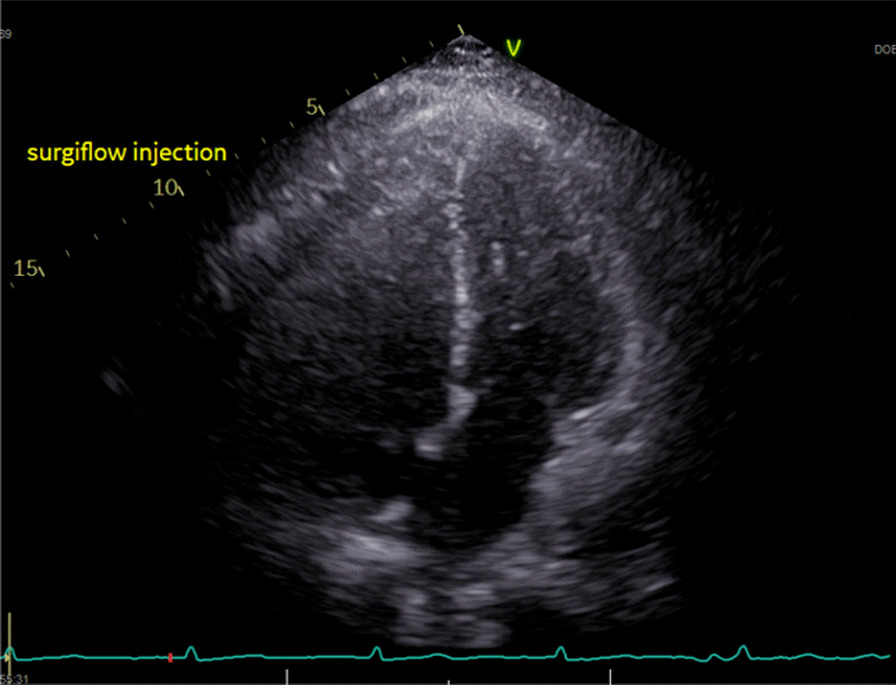
Patient 2 echocardiogram showing SURGIFLO Injection Through the Pericardial Drain. Note the Small but Present Effusion

82 year old female with a BMI of 31.0 and a history significant for diabetes, hypertension, and no smoking history who presented for TAVR with a balloon-expandable valve size 23. Her aortic annulus measurements were as follows: Short axis 21.0 mm, long axis 24.4 mm, area defined effective diameter 22.4 mm, square area 394 mm^2^ (Fig. 2). Of note, her aortic valve was found to have only moderate calcification. She was undergoing a Left femoral approach and valve expansion when she became hypotensive with mean arterial pressure (MAP) as low as 30 mmHg. An emergent echocardiogram was performed, and she was noted to have pericardial effusion. A pericardiocentesis was performed that yielded 200 mL of blood. At that time, SURGIFLO (Johnson & Johnson Wound Management, Somerville, NJ) was injected into the pericardial space to gain hemostatic control (Fig. 3). Repeat aortic root angiography showed no further contrast extravasation. A pericardial drain was left in place for 2 days. Prior to its removal, a chest CT angiogram was obtained which showed no evidence of active extravasation of contrast dye. Her stay was further complicated by atrial fibrillation with rapid ventricular rate, which converted back to normal sinus rhythm with pharmacologic therapy. The remainder of her stay was uncomplicated, and she was discharged home on POD 7. An echocardiogram at one-month post-op showed no effusion and normal valve function.

### Patient 3

**Fig. 4:**
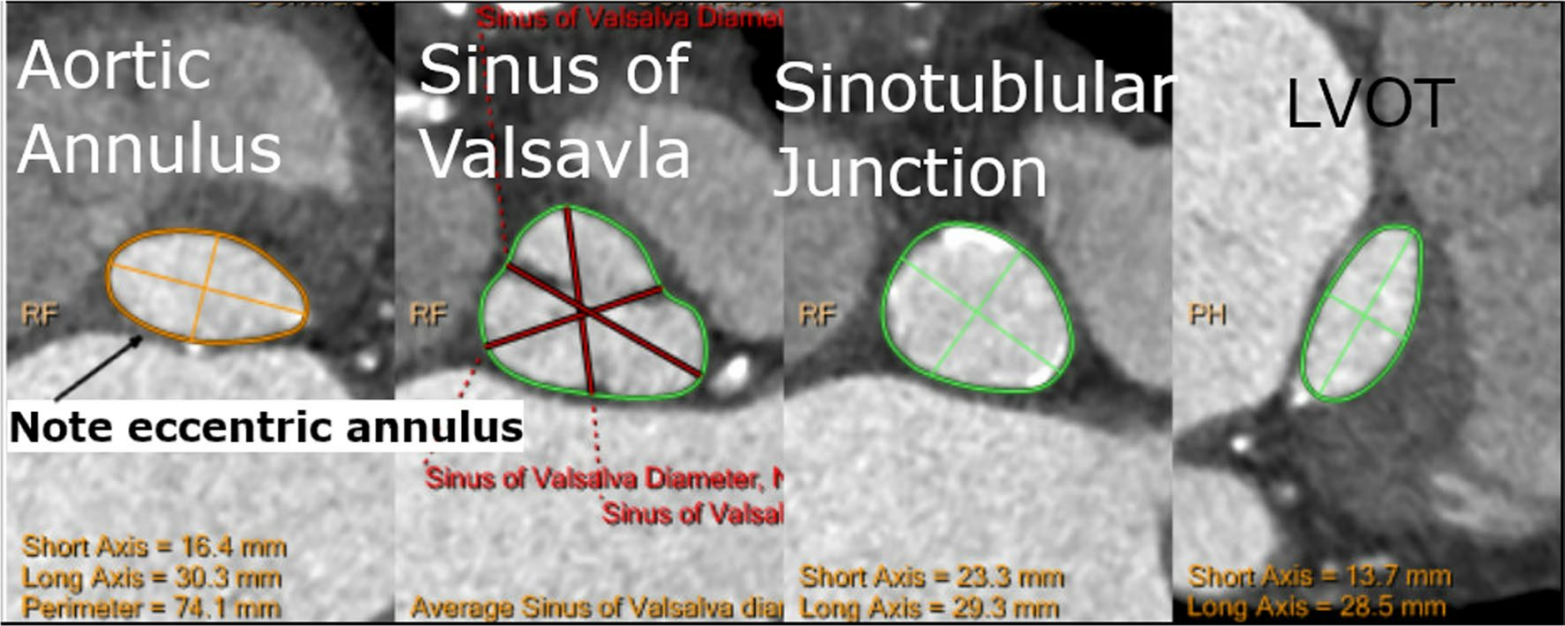
Patient 3 pre-operative CT scan

**Fig. 5:**
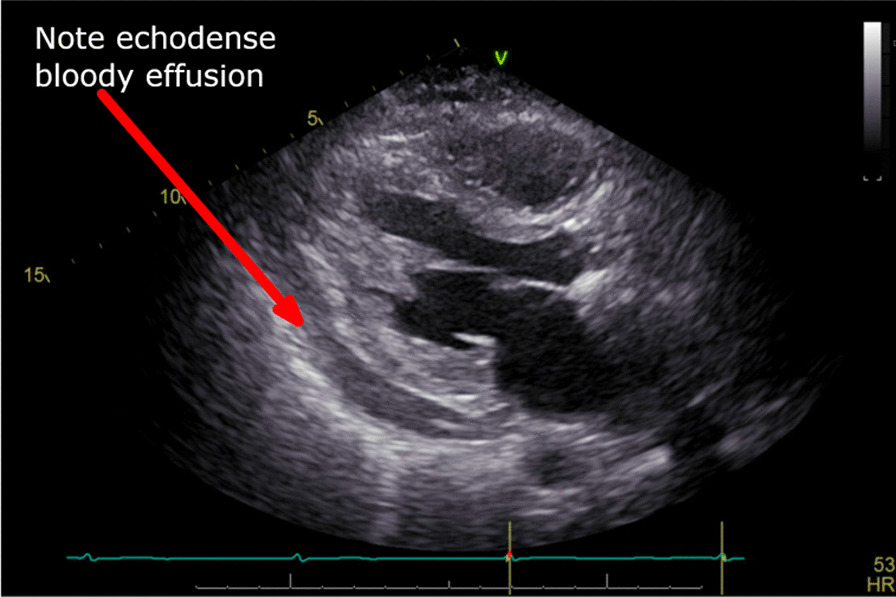
Patient 3 transesophageal echocardiogram showing echo-dense pericardial effusion suggestive of bloody effusion and tamponade

88 year old female with a BMI of 20.4 and a history significant for coronary artery disease, with drug-eluting stent in her right coronary artery, hypertension, hyperlipidemia, and carotid stenosis, as well as osteoporosis, who presented for TAVR with a 23 mm balloon-expandable valve. Her aortic annulus measurements were as follows: Short axis 16.4 mm, long axis 30.3 mm, area defined effective diameter 22.1, square area 383 mm^2^ (Fig. 4). Her aortic valve was found to have significant calcification, with the non-coronary leaflet showing severe calcification and mild calcification of the left coronary leaflet. She underwent a right transfemoral approach TAVR, which was deployed in good position, but initially had a mild to moderate paravalvular leak (PVL). At that time, the decision was made to post-dilate the valve further in an attempt to reduce PVL. Two minutes after the first dilation, the valve was dilated an additional 1 mm in diameter, and the procedure was completed. As the procedure was concluding, about 15 min after the second valve dilation, an echocardiogram was performed, and she was noted to have a new pericardial effusion (Fig. 5). She was re-prepped, and an emergent pericardiocentesis was performed, which yielded 500 cc of blood. She was intubated to facilitate a transesophageal echocardiogram and was diagnosed with a ruptured aortic root. The patient’s healthcare proxy and family declined emergent surgery, and thus a pericardial drain was placed, and arterial blood (determined by oxygen saturation) continued to be aspirated from the pericardial space. In this case, no hemostatic agents of any kind were injected into the pericardial space. Over several minutes, the output, and reaccumulating of blood in the pericardial space slowed, and thus further embolization procedures were deferred. The drain was secured and retained. The patient was admitted to the intensive care unit for conservative management. On post-op day 3, her pericardial drain was removed. Her stay was complicated further by atrial fibrillation with rapid ventricular response, for which she was cardioverted. This was further complicated by a superior cerebellar artery ischemic stroke. She underwent rehabilitation inpatient, and ultimately was discharged on post op day 8 to a skilled nursing facility before returning to her home. One month follow up echocardiogram showed no ongoing pericardial effusion but did show mild to moderate aortic valve regurgitation.

### Common characteristics

All patients were noted to have severe aortic stenosis and calcifications on the aortic valve, ranging from moderate to severe across all leaflets. Two of the patients had somewhat discordant short and long valve axis measurements, suggesting that perhaps valve sizing may be a factor in annular rupture. The ruptures happened at different times throughout the procedures, suggesting that this is a complication providers should be aware of throughout the procedure. Similarly, for two patients, surgical intervention was strongly considered, but ultimately, conservative management was chosen in patients where the valve was already seated at the time of rupture. Notably, the rupture occurred at different points in the procedures for this patient, in line with the findings of Aminian et al. [13]

## Discussion and conclusions

This case series highlights the variability in annular rupture. While some risk factors have been identified, there remain findings on pre-operative imaging that could further classify a patient’s risk. Additionally, these patients suggest that acircular or eccentric valve annuli may contribute to the mechanisms proposed by others, with increased pressure in areas of high calcification. Of note, the short axis in patient 1 and patient 2 exceed the generally accepted upsizing limits of TAVR, while the averaged annular diameter, and long axis do not. Future research should investigate eccentricity of the valve annulus. The authors were unable to find any other studies that have investigated this potential mechanism of annular rupture in any larger studies. Additionally, some consideration of pressure measurement in the balloon should be considered as a mechanism in future research. While these cases did not include pressure measurement during valve deployment, the radial pressure on the calcified valve annulus may play a role in rupture, particularly in valves with unequal calcification across the leaflets. Similarly, further research needs to investigate outcomes of rescue strategies in annular rupture throughout the procedure. If the valve has not been seated yet, for example, appropriate intervention may be different than annular rupture after valve placement, such as in patients 2 and 3. This case series seems to suggest that in rupture after placement, hemostasis can be achieved through conservative management. While not the first case series reporting conservative management of annular rupture, this series further supports the use of conservative medical management of annular rupture and suggests that hemostatic agents may play a role in controlling small leaks in annular rupture. Lastly, this case series suggests that aortic annular rupture can be survivable with rapid identification and timely intervention to prevent a critical hemodynamic collapse.

**Fig. 6:**
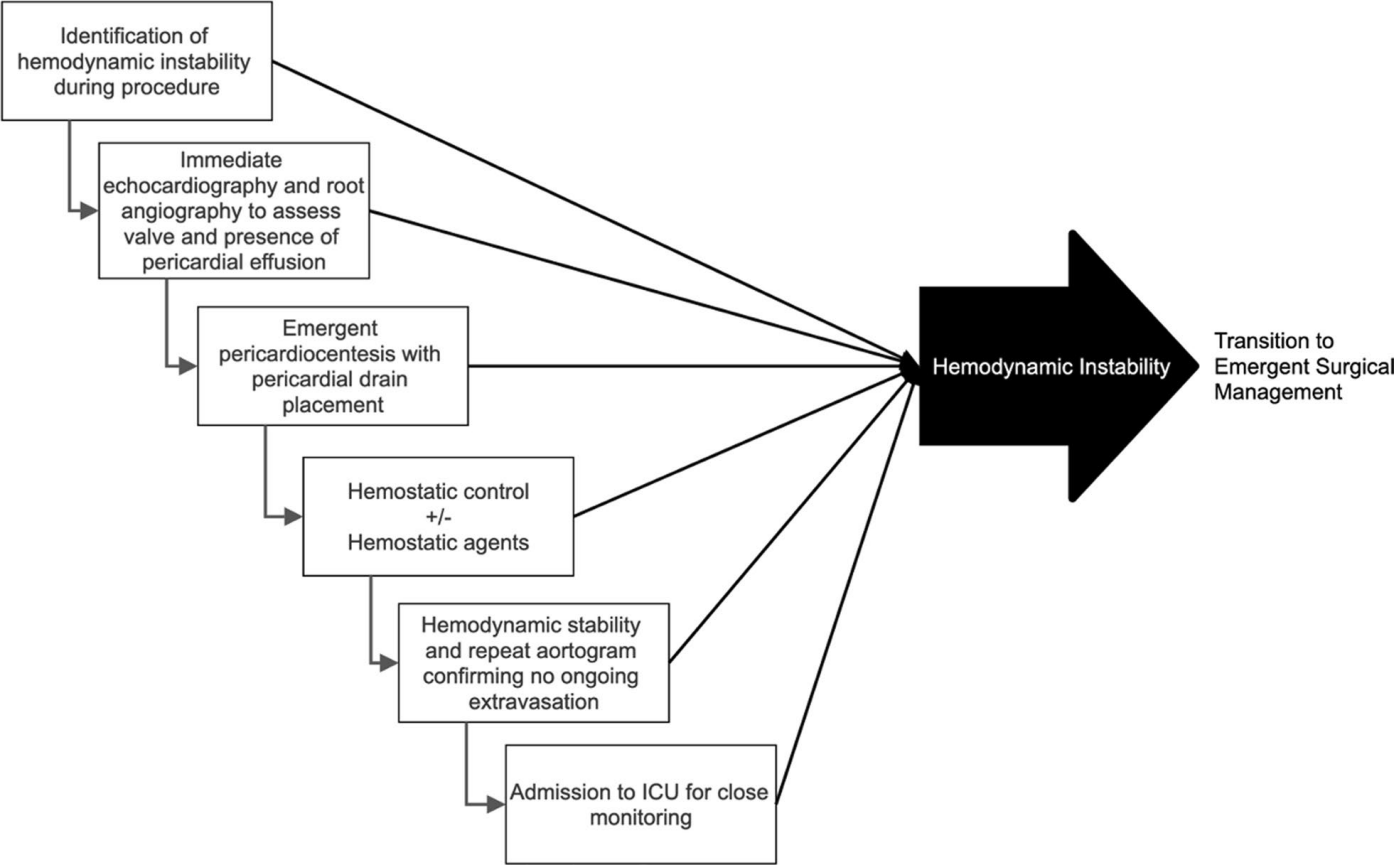
Proposed algorithm for management of annulus rupture during TAVR

We propose the following sequence in the event of potential annular rupture: Identification of hemodynamic instability, an immediate echocardiogram or aortic root angiography to confirm effusion and tamponade physiology, emergent pericardiocentesis and pericardial drain placement for ongoing evacuation of the pericardial space, obtaining hemostasis, follow up aortogram to confirm hemostasis and rule out ongoing extravasation of blood at the aortic root (Fig. 6). If at any time after the pericardiocentesis, hemodynamic instability is observed, it may be reasonable to abandon conservative management and pursue surgical replacement. If, however, the patient’s hemodynamics remain stable, we propose that the decision to proceed to the operating room for surgical management can be deferred until hemostatic control is clearly obtained, or instability re-emerges. ICU monitoring of hemodynamic status is critical in the immediate post-rupture period, allowing for rapid intervention if hemodynamic instability is observed, and close monitoring of drain output to ensure hemostasis. In these patients, it is important that the decision to transition to surgical management be rapid, and decisive if hemodynamic instability persists. Conservative management may be effective for some populations, but we propose that ongoing or repeated hemodynamic instability be the key indicator of the need for surgical intervention in annular rupture. Similarly, standardization of a team-based response through training with a protocolized flow of events may help teams mount a more effective response, and either obtain hemostatic control more rapidly, or make effective, timely decisions to transition to surgical management in this patient population.

## Data Availability

All data generated or analyzed during this study are included in this published article.
